# Biomedical journals and databases in Russia and Russian language in the former Soviet Union and beyond

**DOI:** 10.1186/1742-7622-5-15

**Published:** 2008-09-30

**Authors:** Vasiliy V Vlassov, Kirill D Danishevskiy

**Affiliations:** 1 Dep. Research Methodology, Moscow Medical Academy, Moscow 110000 M. Trubetskaia 7, Russia; 2 Society of Evidence-Based Medicine Specialists, Russia

## Abstract

In the 20^th ^century, Russian biomedical science experienced a decline from the blossom of the early years to a drastic state. Through the first decades of the USSR, it was transformed to suit the ideological requirements of a totalitarian state and biased directives of communist leaders. Later, depressing economic conditions and isolation from the international research community further impeded its development. Contemporary Russia has inherited a system of medical education quite different from the west as well as counterproductive regulations for the allocation of research funding. The methodology of medical and epidemiological research in Russia is largely outdated. Epidemiology continues to focus on infectious disease and results of the best studies tend to be published in international periodicals. MEDLINE continues to be the best database to search for Russian biomedical publications, despite only a small proportion being indexed. The database of the Moscow Central Medical Library is the largest national database of medical periodicals, but does not provide abstracts and full subject heading codes, and it does not cover even the entire collection of the Library. New databases and catalogs (e.g. Panteleimon) that have appeared recently are incomplete and do not enable effective searching.

## Introduction: roots of the paucity of biomedical journals in Russian language

The current state of biomedical journals and databases in Russian language is rooted in the history of the USSR and Russia in 20^th ^century. As with many other aspects of life in the Soviet Union, professional and research training was severely hampered during nearly 70 years of communist rule[1,2]. Some of the major issues impeding biomedical science were the change in style of professional training and the isolation of research groups from the rest of the world.

### Educational system

In earlier Russian history – the 17th century – the educational system was biased towards specialized professional training in applied fields [3]. European style universities in what is now the former Soviet Union came into being in the 19th century [4]. This was followed by a system of educational reforms implemented by the Soviet government in 1930s which increased the number of technical and medical schools, or "institutes," and separated them from the universities [5]. New institutes discontinued teaching research methods and usually did not carry out any research themselves [3]. All research funding was channeled into a separate branch of "research institutes," thus further isolating centers of higher education from research [6].

### Isolationism and hierarchy

In the most glorious days of Russian science, the late 19th – early 20th century, the Russian university educational system was tightly interconnected with that of its European counterparts. It was common for graduates of Russian universities to undertake visiting fellowships and nearly all famous Russian scientists had international experience.

In the1920s, the door was closed from the Soviet side, nearly 30 years before the "official" drop of the "Iron Curtain" [5]. A rigid hierarchy controlling the thoughts and impeding exchange with "ideological opponents" was introduced in both higher education and research, with only a few exceptions. One of exceptions – the Moscow State University, was created under a special category in the USSR national budget, consuming almost 50% of all resources allocated for higher education, and lacked many of the constraints of similar universities thus making it comparable to the European university system. Similar examples could be found for almost every specialty, e.g. the First Moscow Medical Institute (former Medical School of the Moscow University, and currently Moscow Sechenov Medical Academy) became a sort of model medical school with some regulated openness to the west. These model institutions had served "window dressing" function and hence had occasional contacts internationally, while the others existed in complete isolation.

### Thought control in the USSR and the legacy of hierarchical science

A hierarchical system aimed at enforcing the ideology and thought control set up in the early days of the USSR persisted for many years in the field of education. In the field of research, a similar hierarchical system persists even today. One institution (usually in Moscow) per specialty is still nominated as "the leading institution," and its director, "the leading specialist," each yielding great influence over the selection of research priorities as well as the power to settle disputed scientific questions. Previously in the Soviet era, the range of possibilities was determined by the Central Committee of the Communist Party and heads of the "leading institutions" were Party appointees; some of these heads were not prominent scientists [7], but they were still given the power to allocate what insufficient research funding existed between other institutes.

### Biased incentives

The tragedy of the current Russian biomedical science – a consequence of its Soviet past – is that research activities have become an unnecessary exercise for the faculty of Institutes of higher education. It has not been prioritized, not funded, and it is not possible to allocate time for research. For instance, the work load of a non-clinical assistant professor is 800–900 class hours in ten months of the teaching year – i.e. three to four hours six days a week [8]. This regulation is just an example of similar regulations in all universities and schools of vocational training. The workload and the schedule are determined by the school's teaching plan leaving no flexibility for professor and makes research nearly impossible. Traditionally in medical schools in the USSR/Russia professors are hired for a full time job, and therefore they cannot share their working time with research in the research institution.

The only incentive to undertake research, but not to develop a publication record, was – and largely still is – the promotion. The scientific degrees of "candidate of science" (first level) and "doctor of science" (second level) are conditions for the promotion to the associate and full professorship level, respectively. This system of incentives has led to the development of what is known as "dissertational research" – a term officially used by the Higher Attestation Commission (HAC) that supervises and manages the national system of these scientific degrees. Most of the research in institutions of higher education and at least a significant part of research in research institutions became directed to the successful defense of the dissertation. One of the best illustrations of this phenomenon is the total absence of negative results in Soviet science. In 2002 the first author of this paper, in his role as editor of a medical journal, announced a prize of a free subscription to anybody who could provide an example of a medical dissertation with negative results. In two years no single example was offered [9].

Because the dissertation production system became very important and lucrative, strong borders between disciplines were erected and their content was conserved in agreement with the vision of the nominated "leading institutes." An example of this is the field of epidemiology, which continues to be dominated by infectious diseases, resulting in less than ten dissertations on non-infectious disease epidemiology [10] in all of Russia since the beginning of the 21st century.

### Outdated study designs

**Figure 1:**
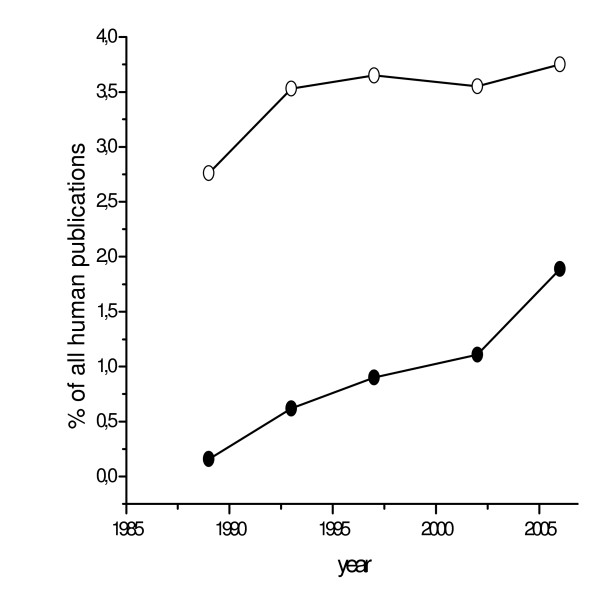
**RCTs as a proportion of all human-subject studies in MEDLINE. **Upper curve – English language publications; bottom line – Russian language publications.

One important aspect of the isolation of Soviet science from the international scientific community was the difficulty in the acceptance or penetration of new research methods. Expensive cohort studies, randomized controlled trials (RCTs) (Figure 1) and case-control studies are still less prevalent in Russia than in Europe and North America. Further, while these study designs are less prevalent in Russian journals as compared with international journals, financial reasons (i.e., high associated costs) alone seem to offer an insufficient explanation. The most obvious reason for the delay in acceptance of new medical research methods is that modern epidemiology is not taught [10]. The terms 'incidence' and 'prevalence' began to be consistently used only since 2000–2005 by some of the more progressive Russian epidemiologists, but some members of the Russian Academy of Medical Science still bluntly reject terms such as "epidemiologic transition" as well as some other basic epidemiological concepts with which they are unfamiliar [11].

## Journals

### The state of the art of the Russian biomedical journals

There are only about 30 Russian journals referenced in MEDLINE (Table 1) and most of them publish abstracts in English, albeit irregularly. Journals referenced in MEDLINE have International Standard Serial Numbers (ISSNs) on their jacket, but the indexing of Russian journals is neither complete nor representative of their totality in any of the existing databases. The ISSN website provides a greater number of periodicals in Hungarian than in Russian, reflecting the degraded state of scientific writing in Russia as well as perhaps some bias on the part of these journals' publishers. To attract submissions, editors pursue the HAC to include their titles among the list of approved periodicals. Authors who publish in these approved journals are permitted to add publications as part of their academic record, and publications for those pursuing scientific degrees (candidate or doctor of science) have become increasingly important. It is illustrative that publications in international journals were not officially taken into account for purposes of receiving academic credit until 2007.

**Table 1:** Russian medical and public health journals referenced in MEDLINE as of 2007

Title	ISSN	Medline abbreviation
AKUSHERSTVO I GINEKOLOGIIA (Obstetrics and Gynecology)	0300-9092	Akush Ginekol (Mosk)
ANESTEZIOLOGIIA I REANIMATOLOGIIA (Anesthesiology and Reanimatology)	0201-7563	Anesteziol Reanimatol
ANTIBIOTIKI I KHIMIOTERAPIIA (Antibiotics and Chemotherapy)	0235-2990	Antibiot Khimioter
EKSPERIMENTALNAIA I KLINICHESKAIA FARMAKOLOGIIA (Experimental and Clinical Pharmacology)	0869-2092	Eksp Klin Farmak
FELDSHER I AKUSHERKA (Feldsher and midwife)	0014-9772	Feldsher Akush
GEMATOLOGIIA I TRANSFUZIOLOGIIA (Haemathology and Transfusiology)	0234-5730	Gematol Transfuziol
GIGIENA I SANITARIIA (Hygiene and Sanitary)	0016-9900	Gig Sanit
GRUDNAIA I SERDECHNO-SOSUDISTAIA KHIRURGIIA (Chest and Cardiovascular Surgery, Continues GRUDNAIA KHIRURGIIA,)	0236-2791	Grud Serdechnososudistaia Khir
KARDIOLOGIIA (Cardiology)	0022-9040	Kardiologiia
KHIRURGIIA (Surgery)	0023-1207	Khirurgiia (Mosk)
KLINICHESKAIA MEDITSINA (Clinical Medicine)	0023-2149	Klin Med (Mosk)
OFTALMOLOGICHESKII ZHURNAL (Journal of Ophthalmology)	0030-0675	Oftalmol Zh
ORTOPEDIIA TRAVMATOLOGIIA I PROTEZIROVANIE (Orthopedics, Traumatology and Prostheses)	0030-5987	Ortop Travmatol Protez
PEDIATRIIA (Paediatrics, Continues SOVETSKAIA PEDIATRIIA)	0031-403X	Pediatriia
PROBLEMY ENDOKRINOLOGII (Problems in Endocrinology, continues PROBLEMY ENDOKRINOLOGII I GORMONOTERAPII)	0375-9660	Probl Endokrinol (Mosk)
PROBLEMY GEMATOLOGII I PERELIVANIIA KROVI (Problems of Haemathology and Transfusiology)	0552-2080	Probl Gematol Pereliv Krovi
PROBLEMY SOTSIAL'NOI GIGIENY, ZDRAVOOKHRANENIIA I ISTORII MEDITSINY (Problems of Social Hygiene, Health Management and History of Medicine)	0869-866X	Probl Sotsialnoi Gig Zdravookhranenniiai Istor Med
PROBLEMY TUBERKULEZA (Problems in Tuberculosis)	0032-9533	Probl Tuberk
STOMATOLOGIIA (Stomatology)	0039-1735	Stomatologiia (Mosk)
TERAPEVTICHESKII ARKHIV (Archive of Internal Medicine)	0040-3660	Ter Arkh
UROLOGIIA I NEFROLOGIIA (Urology and Nephrology)	0042-1154	Urol Nefrol (Mosk)
VESTNIK DERMATOLOGII I VENEROLOGII (Dermatology and Venerology Herald)	0042-4609	Vestn Dermatol Venerol
VESTNIK KHIRURGII IMENI I. I. GREKOVA (Surgical Herald)	0042-4625	Vestn Khir Im I I Grek
VESTNIK OFTALMOLOGII (Ophthalmology Herald)	0042-465X	Vestn Oftalmol
VESTNIK OTORINOLARINGOLOGII (Otorhynolaryngology Herald)	0042-4668	Vestn Otorinolaringol
VESTNIK RENTGENOLOGII I RADIOLOGII (Radiology Herald)	0042-4676	Vestn Rentgenol Radiol
VESTNIK ROSSIISKOI AKADEMII MEDITSINSKIKH NAUK (Russian Academy of Medical Sciences Herald, Continues VESTNIK AKADEMII MEDITSINSKIKH NAUK SSSR)	0869-6047	Vestn Ross Akad Med Nauk
VOPROSY NEIROKHIRURGII (Problems in Neurosurgery, continues ZHURNAL VOPROSY NEIROKHIRURGII IMENI N. N. BURDENKO)	0042-8817	Vopr Neirokhir (Zh Vopr Neirokhir Im N N Burdenko)
VOPROSY ONKOLOGII (Problems in Oncology)	0507-3758	Vopr Onkol
ZHURNAL NEVROPATOLOGII I PSIKHIATRII IMENI S. S. KORSAKOVA (Neurology and Psychiatry Journal, named after Korsakoff)	0044-4588	Zh Nevropatol Psikhiatr Im S S Korsakova

In addition to the above system of approved journals being more weighted than those who have not passed the HAC process, access to existing journals and indeed sometimes their quality is severely limited. Many journals produced by medical professional societies are neither available from their publishers, nor from libraries. Most periodical publications from research institutes and medical schools are not available in medical libraries, except in that of the publishing institution. The content of these "gray" publications is often of a level of quality that would not be acceptable for most Russian journals.

### Biomedical journals in Russia: some explanations of the quality of content

A small number of medical journals were published in Russian during Soviet times, and a negligible number in the languages of other republics of the USSR. Further, these were mostly operated through a "one specialty – one journal" type of system. The chief editor of this journal was usually the leading specialist of the Ministry of Health, who was also heading the leading research institute. In a limited number of disciplines served by more than one prominent institute, up to two journals could have coexisted (e.g. epidemiology and infectious diseases, oncology). Abstracts in Russian journals are generally of low quality, and in most journals they are unstructured and unavailable in English. Only a small number of Russian medical journals make full content available online, but since 2005 some publishers have begun providing electronic versions of their journals on the internet, either independently or collectively through larger projects like E-library.

The content of Soviet medical journals was strictly controlled by editors and censors. Dating back to the Soviet era, most editors did not understand the concept of editorial independence and author responsibility for content and views expressed. Thus, journals are understood by many as a platform for the pronunciation of correct, approved ideas rather than a vehicle that also allows debate or dialogue. For example, because all Soviet journals were registered with the Ministry of Health, studies and comments that criticized decisions of the Ministry of Health were explicitly not accepted by many journals. Nowadays, many journals are linked to medical and professional societies which tend to be run by the same leading specialists or re-registered with publishing houses. Because of highly lucrative publications of pharmaceutical advertisements, successful journals are 'cloned'. For instance, it would not be uncommon to see Chief editor from a respected journal called "*Heart*" to also be Chief editor in similar-themed journals such as "*Cardiology*", "*Heart Complications*", "*Hypertension*", and "*Failure*", and to publish in these journals the content secondary to the "Heart".

Articles published in Russian medical journals can provide a distorted picture, as they cannot claim to lack outside influence. Up to 50% of articles in major Russian journals are connected to advertisements published in the same issue [12]. All journals referenced in MEDLINE claim that they are peer reviewed. It is difficult to concur with or dispute this claim, but the low quality of a significant proportion of articles and the manipulation of content for advertising both give the impression that the peer review process at best is not fully covering content and does not assure the quality of publications. Financial dependence on advertising has certainly distorted the publishing practices of journals. Commercial medical journals are blossoming and thriving, while at the same time traditional medical journals are floundering.

The current state of Russian medical journals, even those linked to professional associations, do not truly reflect the life of the medical community. None of these journals regularly publish medical news, letters and editorials reflecting current problems. Unfortunately, Russian medical journals are slow to accept modern publishing technology, despite enthusiasts making all relevant international guidelines available in Russian [13]. To the best of our knowledge, only one publisher is providing its journal records electronically to the NLM.

### Russian vs. international journals

No international medical journals are published in Russia. The problem has to do with funding: advertisers target only the relevant media, and English language journals appear to be an inappropriate vehicle of information for practicing physicians. Additionally, international journals are not available to even the estimated <5% minority of physicians who can read English [14]. Although the Central Medical Library (CML) in Moscow recently started to subscribe to 371 peer-review journals, these are not available in most other regions. Just as electronic banking is in its early implementation stages in Russia, on-line access to information is also limited.

The total volume of publications in Russia collapsed after *perestroika *(program of large scale societal reforms announced by M. Gorbachev, then Secretary General of the Communist Party of the USSR; this program failed and the USSR dissolved in 1991) and grew afterwards. Since approximately the year 2000, the number of journals and articles in existence is estimated to be higher today than that during Soviet times. Unfortunately, because of the absence of a good registration system and a depressing number of gray journals, it is difficult to obtain reliable estimates.

**Figure 2:**
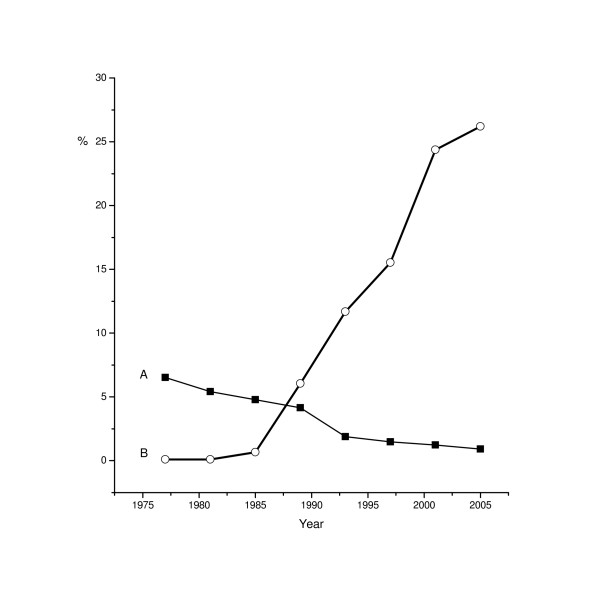
**Proportion of Russian reports reflected in MEDLINE. **A – proportion of reports published in Russian of all reports referenced in MEDLINE; B – proportion of reports with affiliation to Russian institutions of all Russian language plus Russian affiliation reports. Years on abscissa – midyears of three-year intervals.

With liberalization of the Soviet state in the 1980s and increase in international research collaboration, the number of publications from the USSR increased in English language journals. The proportion of methodologically advanced studies 'emigrating' to international English-language journals was comparable to publications of French or German origin[15]. Since *perestroika*, the number of Russian-origin publications in international journals is steadily increasing. Figure 2 illustrates the decreasing proportion of papers in Russian referenced in MEDLINE, while the proportion of papers published by authors with Russian affiliation is increasing. These MEDLINE data should be interpreted with caution, because: (1) the tendencies could be explained by changes in the publications in biomedical research but not clinical or public health research; (2) before 1992 the data present represented all USSR, not only Russia; (3) MEDLINE data reflect the rather conservative set of pre-selected Russian journals; and (4) most of the increase in international publications by Russian scientists has been as a result of the increase in joint publications with foreign coauthors.

Some Russian medical researchers who have been following trends in basic sciences have switched to the well known style of first trying to publish their reports in high impact international journals. This phenomenon is still relatively uncommon in Russian medicine, but the trend is converging with that in Europe. Unfortunately, Russian medical scientists often do not republish their reports in Russian journals. This creates a multi tier system: the best research papers tend to be published in international journals, decent research and studies conducted by recognized professionals are published in a small number of famous Russian journals, and the rest becomes gray literature. One positive trend that may somewhat compensate for this compartmentalization is that many researchers are now turning towards an understanding of science as an international enterprise, and do not limit themselves to exclusively publishing in Russian journals.

A strong tradition of self-referencing in Russian scientific schools, emerging from their isolation, made it unnecessary for the researchers to publish anything outside their *alma mater*. Even as late as the 1990s, some dissertation reviewers counted the number references in Russian, and if there were less Russian papers cited than those in foreign languages, the research degree applicant was criticized as one who lost the patriotism or has insufficient knowledge of the successes of Soviet science. The tradition of self-referencing created the special compartment of Russian language science which is not well reflected by world citation and international databases.

### Public health and epidemiology journals

Journals devoted to epidemiology, preventive medicine, public health and tropical medicine are listed in Table 2 – all are indexed in the CML database. The *Journal of Microbiology, Epidemiology and Immunology *and *Epidemiology and Infectious Diseases *are bastions of infectious diseases epidemiology, and devote most of their page space to clinical and laboratory aspects of infectious diseases. *Social Medicine *is a recent journal devoted to sociological studies of medicine. *Hygiene and Sanitary *is a journal of hygiene in the sense that prevailed in the West before the World War II, that is, it is concerned mostly with issues around food, work place safety, water hygiene, and sanitary surveillance. *Disease Prevention and Health Improvement *is devoted to preventive medicine, public health and health promotion but many of its published papers are methodologically weak. Finally, *The Problems of Social Hygiene, Healthcare and History of Medicine *covers the fields of social hygiene, public health, health management and the history of medicine. It is important to mention that many of the above-mentioned journals suffer from insufficient submission of quality studies and are not necessarily strict about the relevance of the content of articles they accept.

**Table 2:** Russian journals of epidemiology and public health^1^

Title	Published since	ISSN
Zhurnal Mikrobiiologii, Epidemiologii I Immunologii (The Journal of Microbiology, Epidemiology and Immunology)	1923	0372-8714
Epidemiologiia I Infektsionnie Bolezni (Epidemiology and Infectious Diseases)	1996	1560-9529
Sotsiologiia Meditsini (Social Medicine)	2003	17282810
Gigiena I Sanitariia (Hygiene and Sanitary)	1922	0016-9900
Profilaktika Zabolevanii I Ukreplenie Zdorovia (Disease Prevention and Health Improvement)	1961	0022-9040
Problemy Sotsial'noi Gigieny, Zdravookhraneniia i Istorii Meditsiny (The Problems of Social Hygiene, Healthcare and History of Medicine)	1994	0869-866x

^1 ^– As of November 2007 web pages of these journals provide only list of articles, except Profilaktika Zabolevanii I Ukreplenie Zdorovia, where full texts are available for subscribers

## Databases

### History of development of catalogues of Russian biomedical journals

The last attempt to systematize medical publications resulted in a special Catalog of Russian Small Print Publications, published by the State Public Research-Scientific Library (SPRSL) of the USSR in 1987. This Catalog reflected only preprints, conference proceedings and other gray literature in the natural sciences, engineering and medicine. There exists no catalog of Russian medical periodicals. For different purposes, including HAC classification of periodicals, the cataloging system of a subscription agency is used. A somewhat more comprehensive catalog was prepared by the SPRSL, but has not been available since 1990.

The largest catalog of medical publications is supported by the CML, which was transferred to the Moscow Medical Academy in 2001 [16]. However, the CML's catalog is based solely on its own library's collection. During last 20 years, a number of projects attempted to transfer its paper card catalog into a computerized format [17-19] but a large part remains unfinished. The weakness of the CML catalog is its lacunae and its use of the Russian index system. In Soviet times, the Library Bibliographic Classification – an alternative to the commonly used Dewey classification – was developed.

Historically, the Russian CML developed an index for the medical literature which differs from the USA's National Library of Medicine (NLM) index system and its Medical Subjects Headings (MeSH). And although still used by the CML for its published indexes during late Soviet time, it was never completed nor widely accepted. A later attempt at translation to MeSH was mostly unsuccessful because of limited resources, and in 1989 another project aimed to improve this translation was launched in collaboration with NLM. The translation is especially complex for epidemiological terminology and study designs, which often did not exist in the Russian language. A database is currently available on the CML website and while access is free, users need to be aware of the difficulties of using the catalog because understanding the MeSH translation requires a good knowledge of MeSH terms as well as of the older terms used in USSR.

In addition to obstacles connected to the problematic translation of MeSH terms, serious issues exist related to software developed by Russian programmers based on the MARC standard and old ISIS software [20]. This software is employed by many Russian libraries providing internet access, and it is quite different from PubMED. The interface is in Russian and abstracts in English are not available. Interestingly, many libraries charge fees for the assistance of an experienced librarian to help navigate the catalog, as one needs to know the coded terms (which most medics are unfamiliar with) in order to search for relevant literature.

### Recent developments in catalogisation of Russian language biomedical journals

The above-described period of developing electronic catalogs for medical libraries coincided with a period of economic crisis in the USSR and, consequently, in Russia. Libraries of former USSR countries are slowly recovering to a point where they can once again develop their services, including internet access[17,21,22]. The alliance of public scientific libraries [23] is led by the State Russian Library (Moscow), National Russian Library (Saint-Petersburg), and the SPRSL (Moscow). Most of the catalogs do not provide access to the contents of medical journals, and electronic versions of international journals (when available) are password-protected.

The only major database on the post Soviet area, except for those of the Russian libraries mentioned above, is Panteleimon [24]. Panteleimon provides internet access to medical periodicals in Russian and Ukrainian through English, Russian and Ukrainian interfaces. The number of titles of medical journals is impressive, but the number of issues referenced is still small. Public health and epidemiology journals are underrepresented.

Another new project, E-library [25], is referencing 728 biomedical journals, including 192 journals in Russian. Unfortunately, as of 2007, only a couple of issues have been indexed for each journal.

Private and NGO web-based libraries offer only a small number of options that are often of limited interest for researchers conducting literature reviews.

## Internet and Open Access movement

Though subscription is not the major source of income for the publishers of Russian journals, they do not make the content of their journals freely accessible online. None of the journals are published on the principles of Open Access (OA), despite the idea of OA being well presented in Russian, e.g. thanks to support offered by George Soros (Budapest Open Access Initiative, 2001) and later publications [26]. The only OA journal to be registered in Russian – Health Management [27] has ceased publication.

The obvious barrier to starting an OA journal is the absence of non-binding funding sources. Leading specialists first attempt to start print journals in order to attract advertising and academic recognition by applying to be included on the HAC list of recognized journals.

## From Closeness to One World

### "Iron curtain" dismantled?

With liberalization following the fall of communism in Russia, philanthropist George Soros championed support for Russian scientists in the form of grants. Later the state system of grant support for research (Russian Fundamental Studies Foundation and Russian Humanitarian Studies Foundation) was introduced, now commonly used by scientists, but it provides very limited funding [28]. However, even this system is strongly influenced by specialists from "leading" institutes, notably those from the Soviet past. Much of current research funding is still distributed through ministerial bureaucratic and sometimes patronizing mechanisms, and in more recent years through possibly even less transparent "national projects."

In many fields of research and especially in medicine, industry sponsorship has played a dubious role. On one hand, industry was the first to raise the notion of research methodology in the biomedical field, e.g. through introduction of international drug trials, especially since the 1980s. On the other hand, drug and medical device producers penetrated science from the leading research institutes, such as the National Cancer Centre, all the way down to provincial medical institutes. As a result, professors frequently deliver conference lectures using industry provided presentations; "scientific" sessions are given with undisclosed industry sponsorship; halls of medical schools are decorated by drug company propaganda. Publications of so called "opinion leaders" in journals are routinely prepared by the pharmaceutical companies.

### Barrier of language

Since Soviet Russia had been isolated from the western world, only ideologically-reliable communist scientists were allowed to travel abroad. Even for them, a short trip to Europe or the USA occasionally led to imprisonment for five to ten years for betrayal or espionage. The flow of Russian research articles to international journals was exhausted before World War II, because publication in such journals could be perceived to demonstrate a preference for the foreign (i.e., non-Soviet) platform. Even papers about successes of Soviet science that were officially commissioned from the author and approved by propaganda officials for publication in international journals could lead to persecution [1].

The teaching of foreign languages in schools before as well as after World War II was motivated by the need for communication with prisoners of war. However, self training in foreign languages could have been a sufficient cause for imprisonment in deadly Siberian camps [29]. In institutes of higher education, language classes were considered useless because contacts with foreign specialists and access to journals were very limited. The largest foreign literature collections that appeared in the USSR were captured from Germany and its allies in 1945. After this, most institutes of higher education had no access to international journals. A small number of the best journals – such as *Science *and *Nature *– were reprinted (without obtaining any permission from the publishers) but with severely censored content. Since the 1970s and with the influx of oil money, the Soviet government purchased a small number of journal subscriptions for the Academy of Sciences and the largest libraries, but this small funding source dried out in the mid-1980s.

During the Soviet era, Russian was the only scientific language, e.g. in addition to Latin, terminology in anatomy Russian was used. After the collapse of the USSR, the newly independent countries went different ways, with some embracing English terminology (Baltic countries and Georgia, personal communications, A. Erenberg, L. Zaalishvilli), while others continued to use Russian (Central Asia, Azerbaijan, and Byelorussia, personal communications, J. Sadikova) or tried to create a national scientific language in addition to English (Ukraine, personal communications, E. Telischevska).

Medical specialists in Russia today strongly depend upon the information obtained from handbooks, advertisements and even the drug representatives themselves[30]. The inclination of Russian physicians to have a good reliable textbook, often used throughout one's professional life, provides several publishing houses with a guaranteed, commercially successful product. Unfortunately, no regularly updated information sources are available to Russian physicians except for the Cochrane Reviews' abstracts and some other free English language materials. Because an estimated 95% of doctors in Russia are unable to read in English[14], even these limited resources are underused.

## Are we stuck with our "Great Soviet science"?

From the Soviet era there exists a myth – still widely accepted by the public and many in the professional community – that Russian science is the leader in the world, at least in the major strategic areas (as well as in education and healthcare in general). Some criticism is permitted today, but only in relation to current problems and provided it contains immediate reference to the glorious successes of the past. A number of senior academicians use the argument – popular in Soviet times and used as propaganda for Communist ideas – that the Semashko system is the best in the world and on its basis call for a resurrection of the Soviet model of healthcare.

In connection with the self-presentation of Soviet science, there were a couple of efforts to publish scientific journals in English with collections of the best articles, but all of these projects were short-lived. At the same time, competitive Soviet journals (mostly in aerospace technology, chemistry, physics) were copyrighted by major Western publishers and translated and have been at least partially available in English since the 1960s.

While the medical research methodology in Russia is progressing in recent years, albeit very slowly there is clear need to create better incentives structures for publishing as well as to deal with the language barrier in order to overcome the Soviet legacy of isolation and ideology. Greater acceptance of critical thinking and improvement of transparency of scientific conduct seem to be the only existing driving forces for change.

## Summary

• The state of the biomedical literature in Russian is drastic for a clear set of historical reasons: such as communist ideology and isolation of the USSR from the international domain. Currently perverse structure of incentives and language barrier impede improvement.

• The medical research methodology in Russia is progressing in recent years, albeit very slowly. Low validity of many studies undertaken in Russia makes them irrelevant for systematic reviews.

• Medical journals are far behind the international practice in promoting editorial independence, adopting peer-review processes and other standards of modern scientific publishing.

• The library based biomedical databases are mostly not available online, are old-fashioned and do not enable effective search.

• Open access continues to be a far horizon in the development of biomedical publishing in Russia.

• There is an urgent need to improve teaching of research methods, and to improve libraries. Greater emphasis is needed on teaching English, as well as making English a prerequisite of medical profession.

## Abstracts in alternative languages

The abstract of this editorial has been translated into the following languages by the following translators (names in brackets):

• Chinese – simplified characters (Mr. Isaac Chun-Hai Fung) [see Additional file 1]

• Chinese – traditional characters (Mr. Isaac Chun-Hai Fung) [see Additional file 2]

• French (Ms. Annick Borquez) [see Additional file 3]

• Russian (The authors) [see Additional file 4]

• Spanish (Ms. Annick Borquez) [see Additional file 5]

## Competing interests

The authors declare that they have no competing interests.
